# Supplementary material for: Electrocatalytic water oxidation with manganese phosphates
Source: Nat Commun. 2024 Feb 15;15:1410. doi: 10.1038/s41467-024-45705-1 (PMC10869713; doi:10.1038/s41467-024-45705-1)
Supplement: Supplementary file 1 — Supplementary Information [file 41467_2024_45705_MOESM1_ESM.pdf]

Supplementary Information

Electrocatalytic Water Oxidation with Manganese Phosphates

Authors: Shujiao Yang[1#], Kaihang Yue[2#], Xiaohan Liu[1], Sisi Li[1], Haoquan Zheng[1], Ya Yan[2*], Rui Cao[1], and Wei Zhang[1*]

Affiliations:

[1]Key Laboratory of Applied Surface and Colloid Chemistry, Ministry of Education; School of Chemistry and Chemical Engineering, Shaanxi Normal University, Xi'an 710119, China.

[2]Shanghai Institute of Ceramics, Chinese Academy of Sciences (SICCAS), Shanghai 200050, China.

*Corresponding authors. Emails: zw@snnu.edu.cn (W.Z.); yanya@mail.sic.ac.cn (Y.Y.).

#These authors contributed equally: Shujiao Yang, Kaihang Yue

General materials

$MnCl_2$•$4H_2O$ (99.0%, Alfa), K_2HPO_4 (99.0%, Energy Chemical), $Na_4P_2O_7$•$10H_2O$ (99.0%, Energy Chemical), NaH_2PO_4•$2H_2O$ (99.99%, Alfa), Na_2HPO_4•$12H_2O$ (99.99%, Alfa), C_3H_6O (99.5%, Alfa), CH_3CN (99.8%, Sinopharm Chemical), Nafion (5 wt%, DuPont), Na_2SO_4 (99.0%, Alfa), $H_2{}^{18}O$ (99.0%, Alfa), *n*-Bu_4NPF_6 (98%, Energy Chemical) were attained from commercial suppliers without further purification. Milli-Q water (18.2 MΩ•cm) was used in all experiments.

Supplementary Table 1 The Bader charge analysis of the Mn(IV)−O* state for $KMnPO_4$.

Atom	X	Y	Z	Charge	Min distance	Atomic vol	Bader charge
O1	3.460	1.877	8.494	7.496	0.790	17.455	1.496
O2	1.451	3.957	4.365	7.490	0.798	14.524	1.490
O3	3.585	2.934	1.880	7.501	0.773	18.058	1.501
O4	2.462	1.763	4.857	7.494	0.789	17.045	1.494
O5	0.003	1.892	4.410	7.486	0.761	16.775	1.486
O6	0.932	2.827	6.548	7.483	0.796	17.089	1.483
O7	0.201	2.085	0.626	7.489	0.794	17.297	1.489
O8	4.134	0.512	1.611	7.506	0.808	17.372	1.506
O9	2.017	6.930	0.820	7.496	0.790	17.456	1.496
O10	4.026	4.850	4.949	7.491	0.798	14.527	1.491
O11	1.892	5.873	7.434	7.501	0.773	18.058	1.501
O12	3.015	7.044	4.458	7.494	0.789	17.045	1.494
O13	5.474	6.915	4.904	7.486	0.761	16.773	1.486
O14	4.545	5.980	2.767	7.483	0.796	17.089	1.483
O15	5.276	6.722	8.689	7.489	0.794	17.297	1.489
O16	1.374	8.296	7.704	7.506	0.808	17.371	1.506
P1	4.202	1.888	0.956	1.334	0.468	3.349	−3.665
P2	4.285	6.213	4.257	1.349	0.469	3.342	−3.650
P3	1.275	6.919	8.358	1.334	0.468	3.349	−3.666
P4	1.192	2.595	5.057	1.349	0.469	3.342	−3.650
K1	4.166	8.747	6.474	8.125	1.231	20.479	−0.875
K2	4.284	4.414	7.581	8.131	1.235	21.139	−0.869
K3	1.311	0.060	2.841	8.125	1.231	20.479	−0.875
K4	1.193	4.393	1.734	8.131	1.235	21.138	−0.869
**Mn1**	3.786	3.084	3.907	11.548	0.880	12.374	**−1.452**
**Mn2**	3.982	7.236	1.281	11.567	0.873	13.506	**−1.433**

Vacuum charge: 0 Vacuum Volume: 0 Number of Electrons: 204

Supplementary Table 2 The Bader charge analysis of the Mn(IV)−O* state for $KMnPO_4 \bullet H_2O$.

Atom	X	Y	Z	Charge	Min distance	Atomic vol	Bader charge
O1	1.864	4.727	3.670	7.486	0.824	14.278	1.486
O2	3.168	2.508	1.218	7.479	0.825	14.056	1.479
O3	4.683	2.508	1.218	7.480	0.798	14.105	1.480
O4	0.324	4.727	3.670	7.488	0.811	14.117	1.488
O5	2.341	5.129	1.475	7.464	0.843	15.002	1.464
O6	2.691	6.920	3.239	7.503	0.843	17.7110	1.503
O7	5.179	1.674	3.542	7.335	0.655	17.105	1.335
O8	5.530	2.105	3.928	7.473	0.827	15.019	1.473
O9	1.097	0.315	0.786	7.496	0.815	18.139	1.496
O10	1.097	5.219	1.055	7.317	0.658	15.980	1.318
P1	1.103	5.423	2.998	1.412	0.494	3.607	−3.588
P2	3.935	1.812	0.545	1.419	0.497	3.568	−3.581
K1	1.097	8.095	0.765	8.137	1.280	20.241	−0.863
K2	3.935	7.476	3.218	8.136	1.267	20.596	−0.864
**Mn1**	1.097	3.473	0.211	11.511	0.904	10.657	**−1.488**
**Mn2**	3.935	3.762	2.663	11.495	0.904	10.253	**−1.504**

Vacuum charge: 0 Vacuum Volume: 0 Number of Electrons: 118

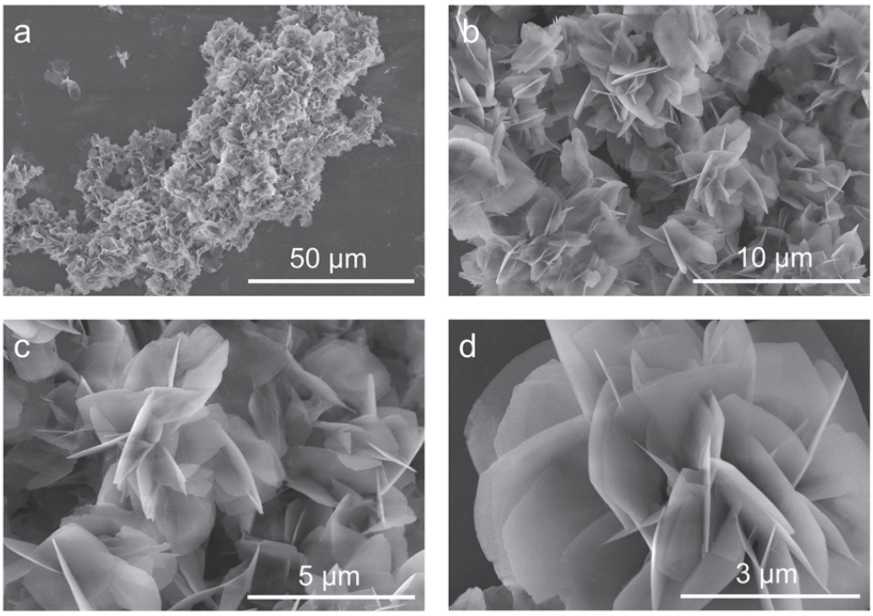

Supplementary Fig. 1 The SEM images of the $KMnPO_4$•H_2O catalyst with (a) 1.00 k, (b) 5.00 k, (c) 10.0 k, (d) 18.0 k magnifications.

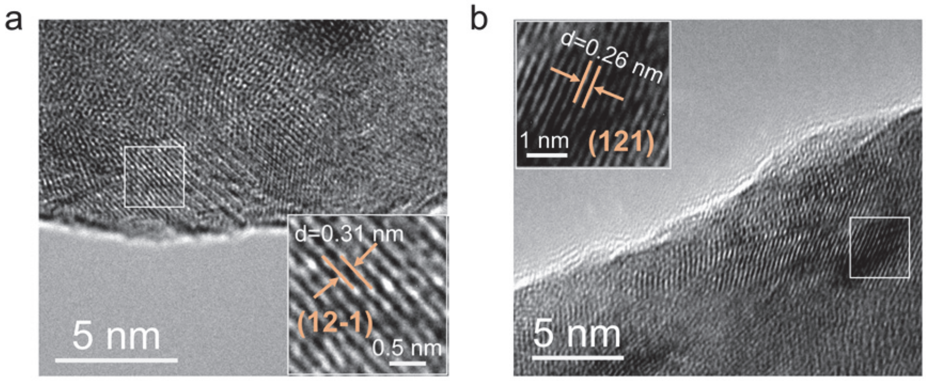

Supplementary Fig. 2 The HR-TEM images of the (a) $KMnPO_4$ and (b) $KMnPO_4 \cdot H_2O$ catalysts.

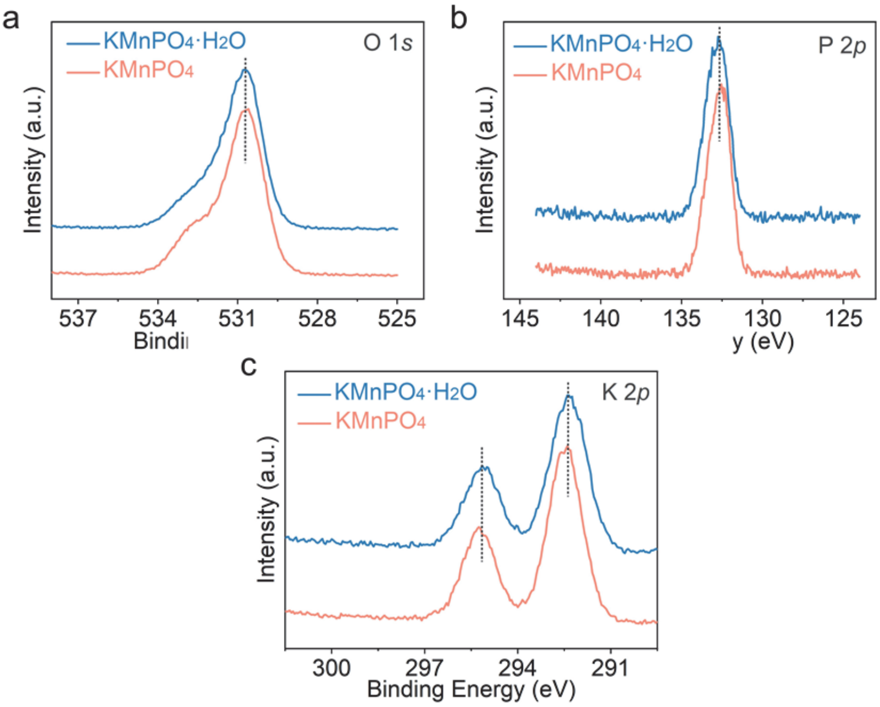

Supplementary Fig. 3 The XPS spectra of $KMnPO_4$•H_2O and $KMnPO_4$ at (a) O 1*s*, (b) P 2*p*, and (c) K 2*p* regions.

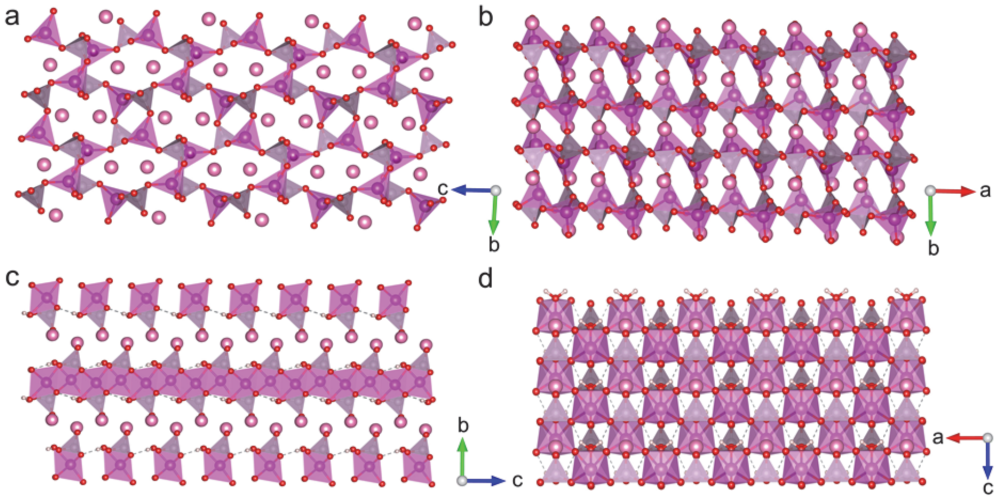

Supplementary Fig. 4 The crystal structures of (a, b) $KMnPO_4$ and (c, d) $KMnPO_4•H_2O$.

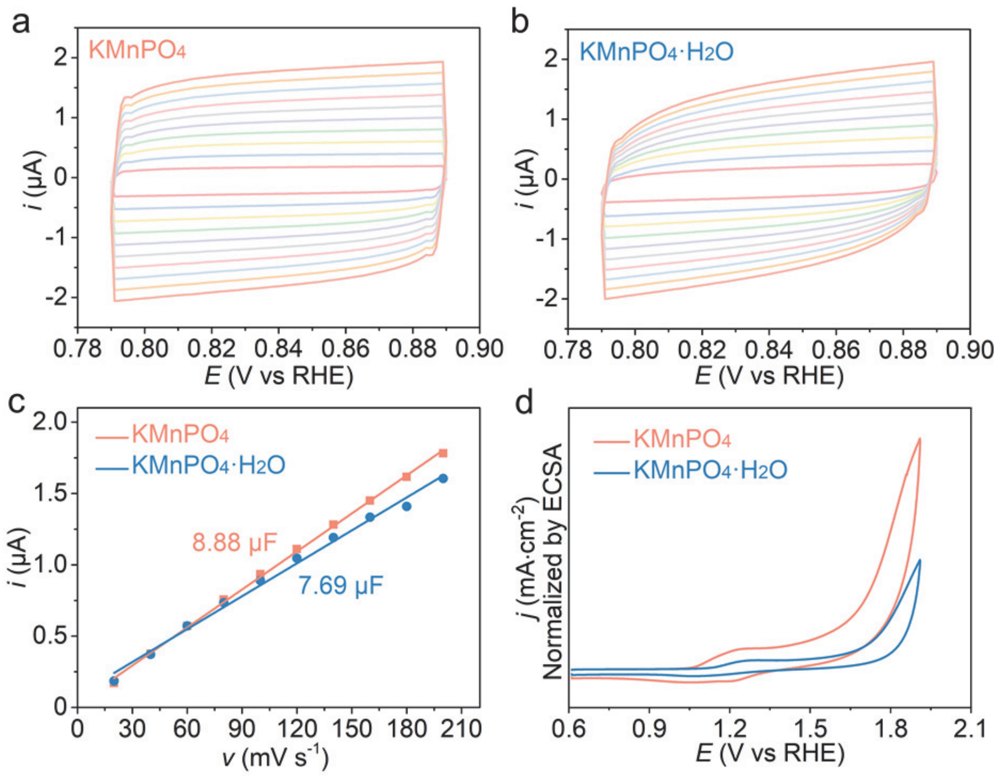

Supplementary Fig. 5 The charge-discharge currents of (a) $KMnPO_4$ and (b) $KMnPO_4$•H_2O estimated in the non-Faradaic potential range with scan rates from 20 to 200 mV•s^{-1}. (c) The relationship between the anode charging current and the scan rates at 0.84 V of $KMnPO_4$ and $KMnPO_4$•H_2O; the capacitance value is proportional to the electrochemical surface area (ECSA). (d) The normalized OER performances by ECSA of the $KMnPO_4$ and $KMnPO_4$•H_2O catalysts.

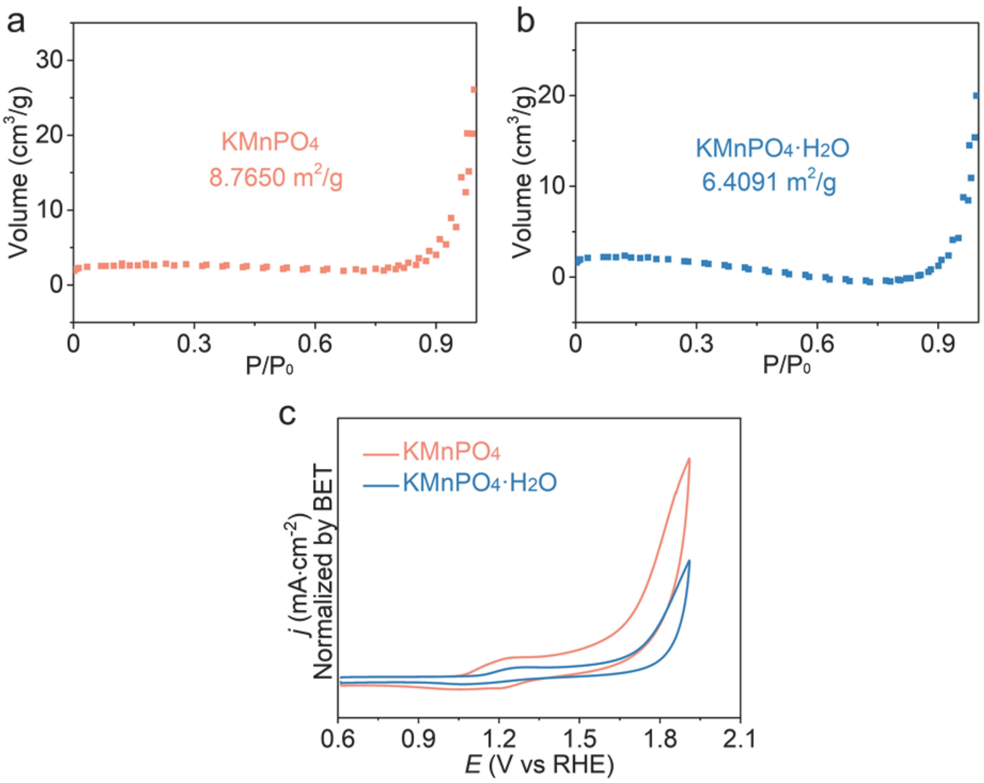

Supplementary Fig. 6 The N_2 adsorption-desorption curves of (a) $KMnPO_4$ and (b) $KMnPO_4$•H_2O. (c) The normalized OER performances by BET surface areas of the $KMnPO_4$ and $KMnPO_4$•H_2O catalysts.

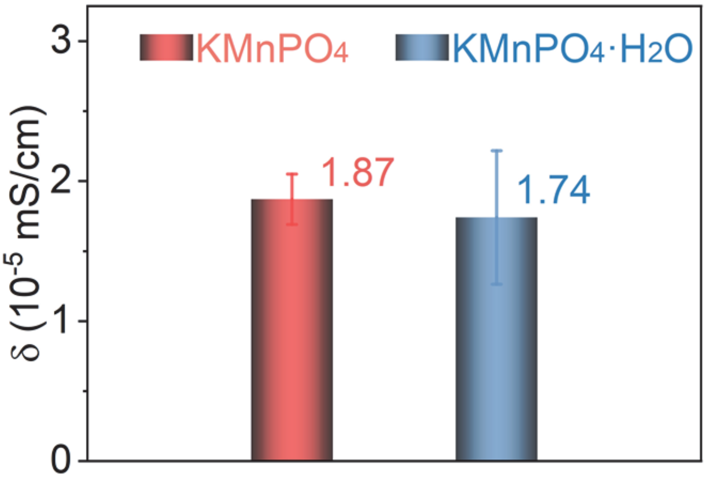

Supplementary Fig. 7 The conductivity of the $KMnPO_4$ and $KMnPO_4$•H_2O catalysts.

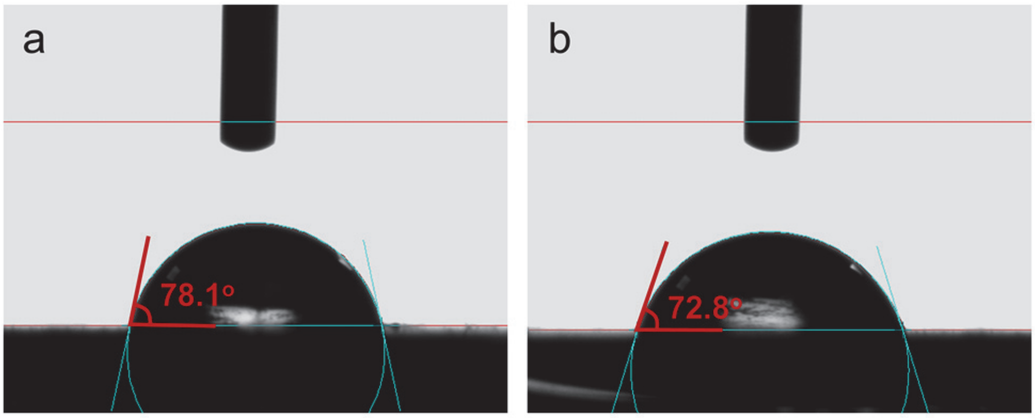

Supplementary Fig. 8 The contact angles of water droplets of the (a) $KMnPO_4$ and (b) $KMnPO_4•H_2O$ catalysts.

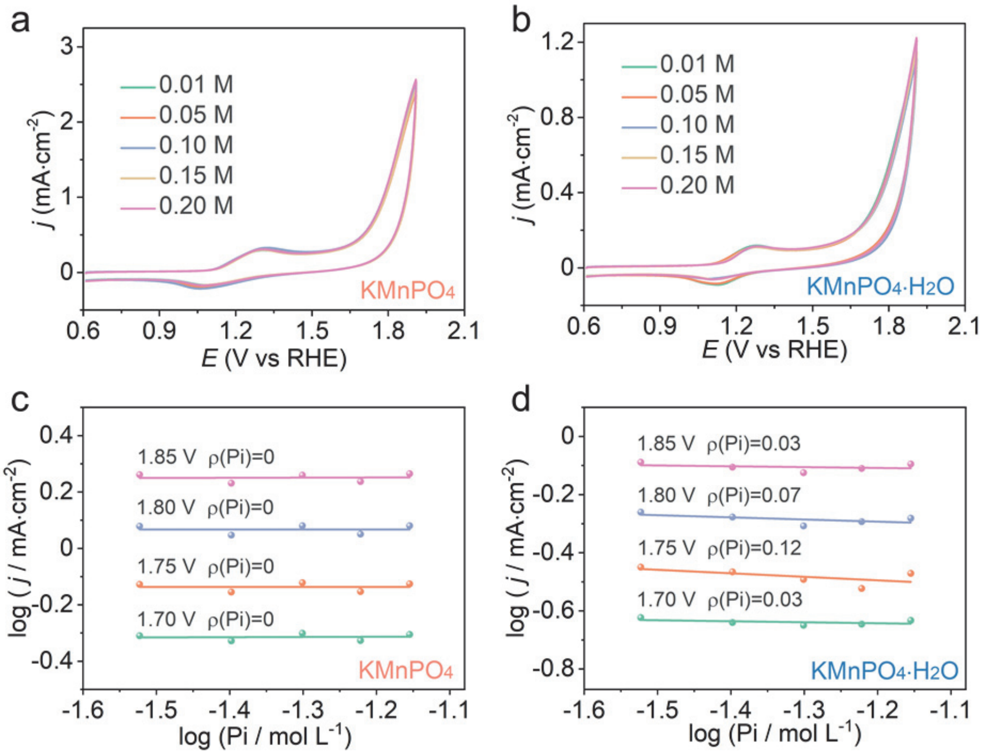

Supplementary Fig. 9 (a, b) The current response of the $KMnPO_4$ (a) and $KMnPO_4$•H_2O (b) catalysts to the concentration of phosphates. (c, d) The phosphate concentration dependence of the catalytic currents of the $KMnPO_4$ (c) and $KMnPO_4$•H_2O (d) catalysts in phosphate buffer with different concentrations at the potential range of 1.70 V-1.85 V. The ionic strength is adjusted to be the same in the electrolytes using Na_2SO_4.

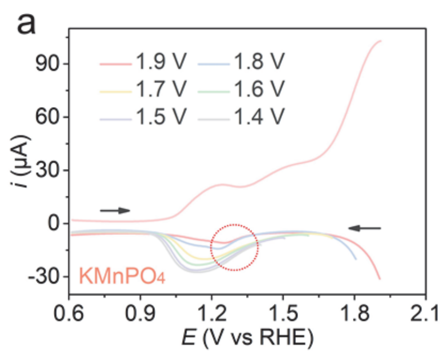

Supplementary Fig. 10 Square wave voltammogram polarization curves of (a) $KMnPO_4$ and (b) $KMnPO_4$•H_2O in 0.05 M PBS solution (pH=7.0) in the positive and negative directions after setting the electrode for 30 s at a certain potential.

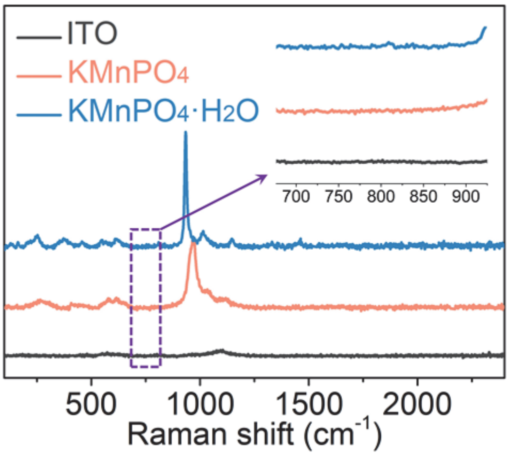

Supplementary Fig. 11 The Raman spectra of the $KMnPO_4$, $KMnPO_4$•H_2O, and blank ITO electrode.

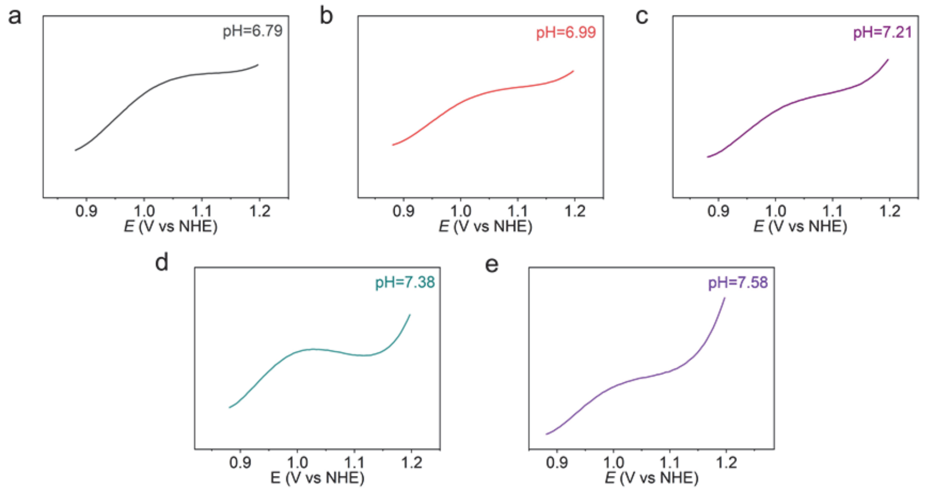

Supplementary Fig. 12 The precatalytic $Mn^{III/IV}$ oxidation events from DPV of $KMnPO_4$ in electrolytes with (a) 6.79, (b) 6.99, (c) 7.21, (d) 7.38, (e) 7.58 different pH values.

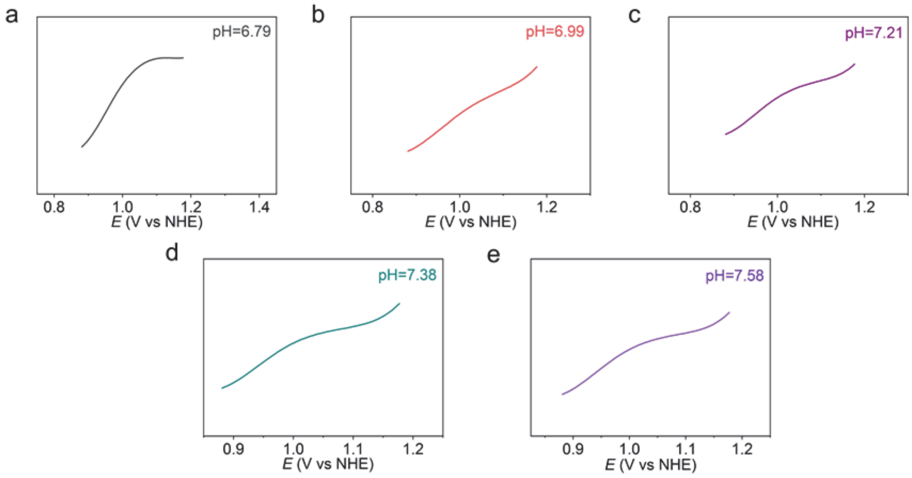

Supplementary Fig. 13 The precatalytic $Mn^{III/IV}$ oxidation events from DPV of $KMnPO_4$•H_2O in electrolytes with (a) 6.79, (b) 6.99, (c) 7.21, (d) 7.38, (e) 7.58 different pH values.

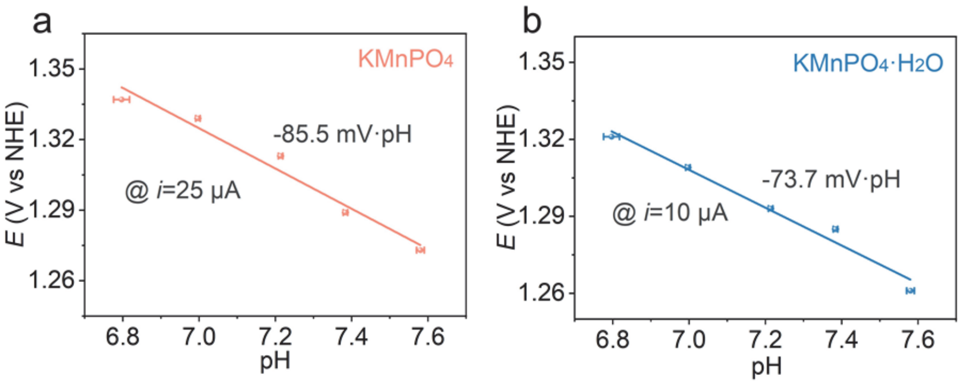

Supplementary Fig. 14 The potential responses of the (a) $KMnPO_4$ and (b) $KMnPO_4$•H_2O to the pH values of the electrolyte from SWV in Figs. 4h-4i.

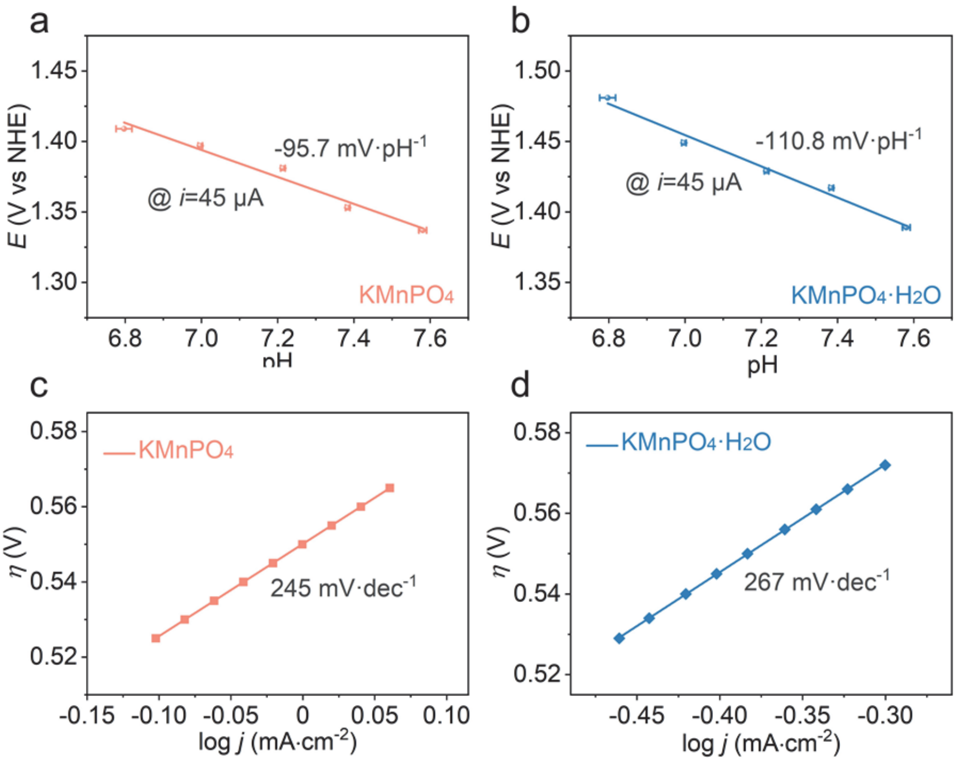

Supplementary Fig. 15 (a, b) The potential responses of the two electrocatalysts to the pH values of the electrolyte at i = 45 μA from SWV in Figs. 4h-4i. (c, d) Tafel plots of the two electrocatalysts for OER.

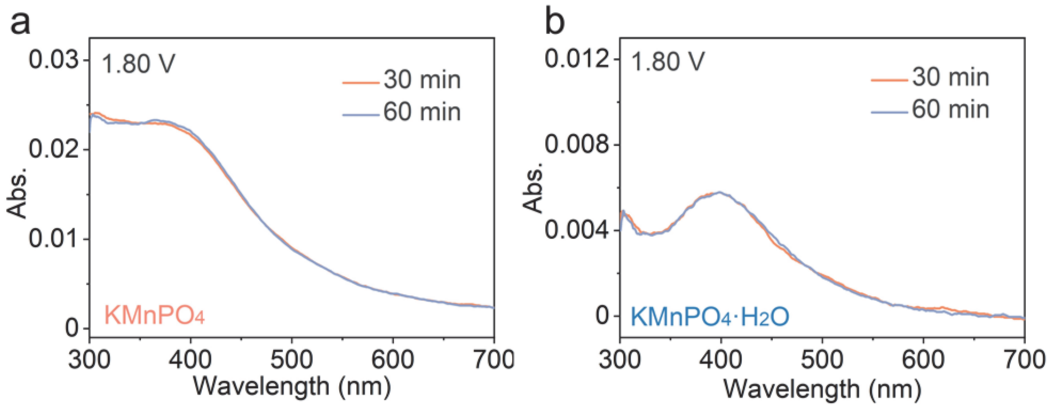

Supplementary Fig. 16 The in-situ time-dependent UV-vis spectra of the (a) $KMnPO_4$ and (b) $KMnPO_4 \cdot H_2O$ at 1.80 V.

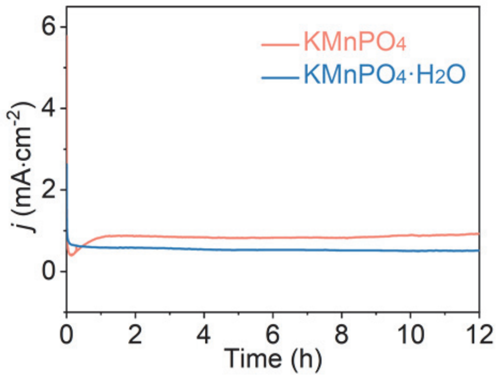

Supplementary Fig. 17 The controlled potential electrolysis of the two catalysts for OER at 1.70 V without iR compensation.

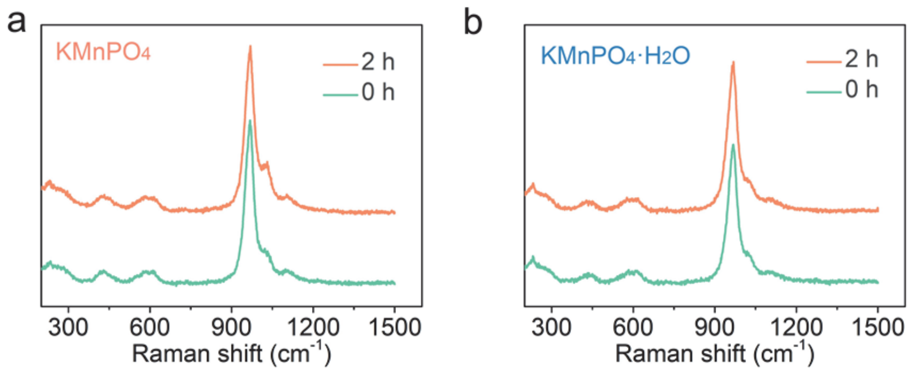

Supplementary Fig. 18 The Raman spectra of the (a) $KMnPO_4$ and (b) $KMnPO_4$•H_2O after electrolysis for 2 h at 1.70 V.

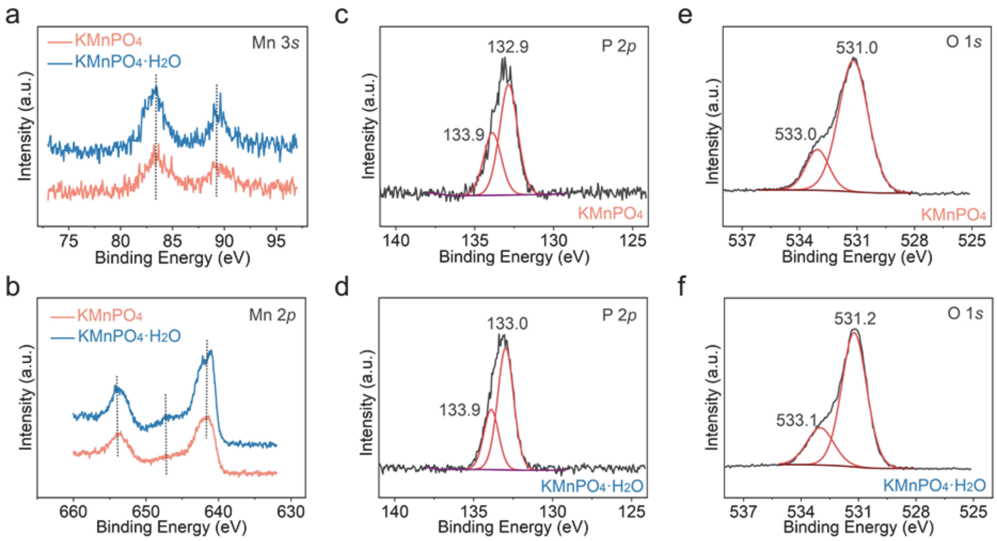

Supplementary Fig. 19 The XPS spectra of (a) Mn 3*s* and (b) Mn 2*p* of $KMnPO_4$ and $KMnPO_4•H_2O$ after OER electrolysis. The XPS spectra of P 2*p* and O 1*s* of (c, e) $KMnPO_4$ and (d, f) $KMnPO_4•H_2O$ after OER electrolysis.

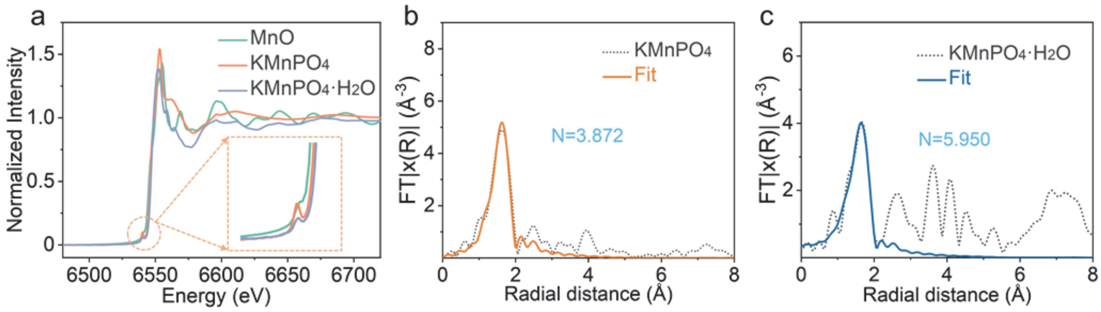

Supplementary Fig. 20 (a) Ex-situ XANES spectra for Mn K-edge of $KMnPO_4$ and $KMnPO_4$•H_2O. (b, c) The FT-EXAFS fitting curves at R space for Mn K-edge of $KMnPO_4$ (b) and $KMnPO_4$•H_2O (c).

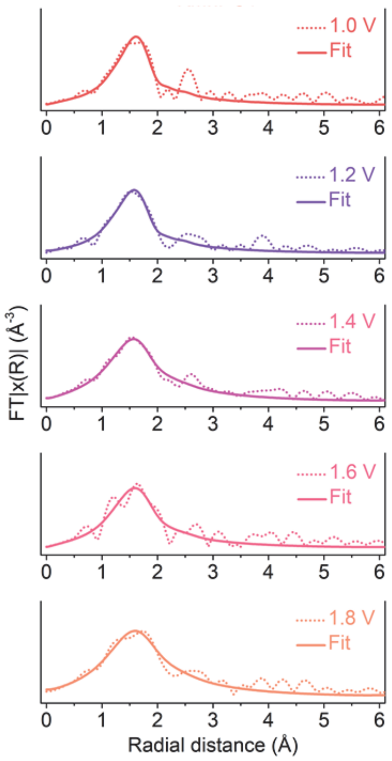

Supplementary Fig. 21 The FT-EXAFS fitting curves at R space of Mn K-edge for $KMnPO_4$.

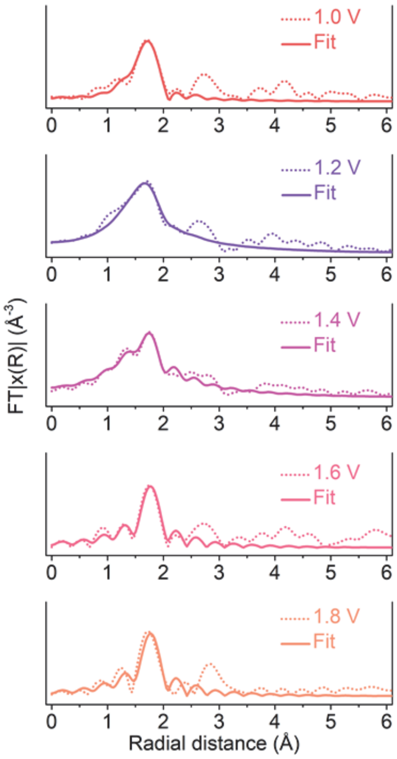

Supplementary Fig. 22 The FT-EXAFS fitting curves at R space of Mn K-edge for $KMnPO_4$•H_2O.

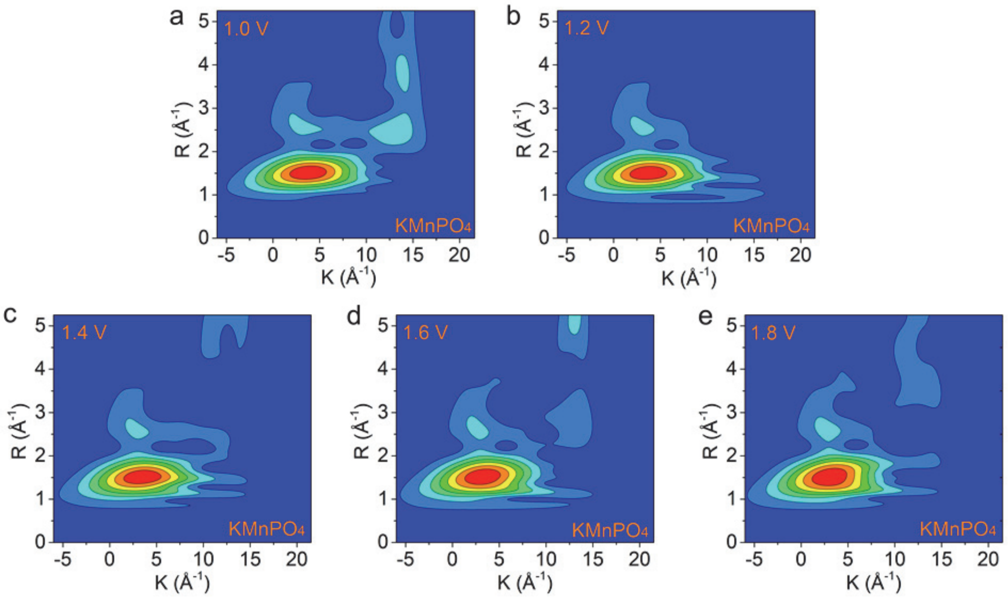

Supplementary Fig. 23 The WT-EXAFS plots of the $KMnPO_4$ catalyst at (a) 1.0 V, (b) 1.2 V, (c) 1.4 V, (d) 1.6 V, (e) 1.8 V different potentials.

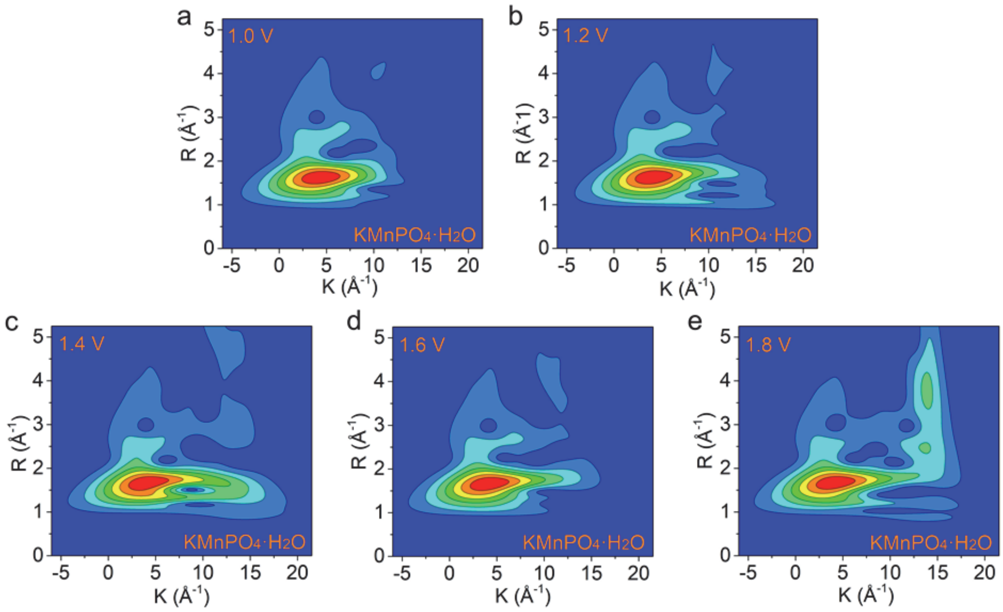

Supplementary Fig. 24 The WT-EXAFS plots of the $KMnPO_4 \cdot H_2O$ catalyst at (a) 1.0 V, (b) 1.2 V, (c) 1.4 V, (d) 1.6 V, (e) 1.8 V different potentials.

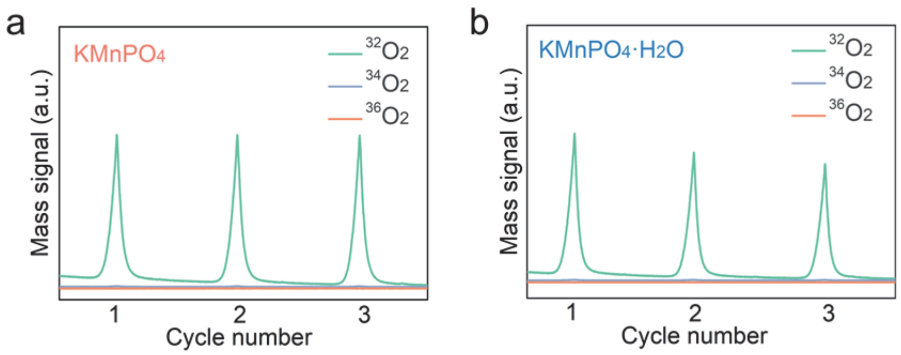

Supplementary Fig. 25 The DEMS signals of $^{32}O_2$, $^{34}O_2$, and $^{36}O_2$ from the gaseous products of $KMnPO_4$ (a) and $KMnPO_4$•H_2O (b) catalysts in $H_2{}^{16}O$ aqueous PBS electrolyte during three times of cycles in the potential range of 0.90 to 2.10 V at a scan rate of 5 mV•s^{-1}.

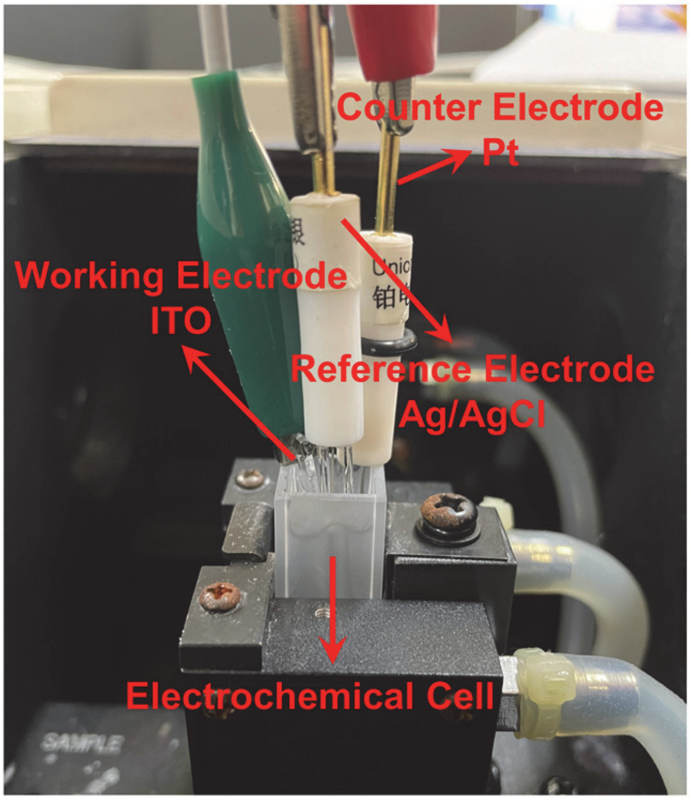

Supplementary Fig. 26 Equipment for in-situ UV-vis absorption spectra test.

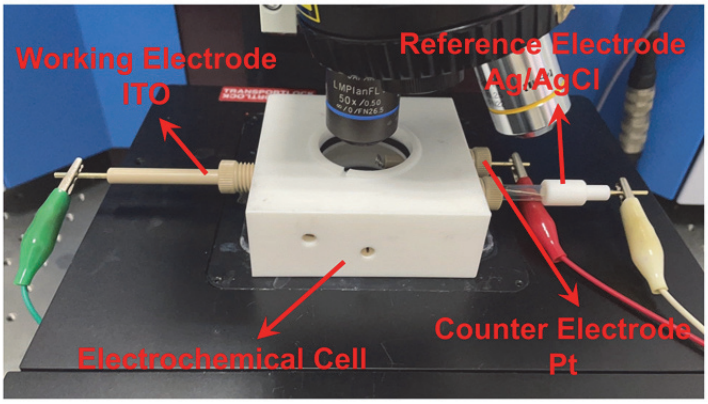

Supplementary Fig. 27 Equipment for in-situ Raman spectra test equipment.

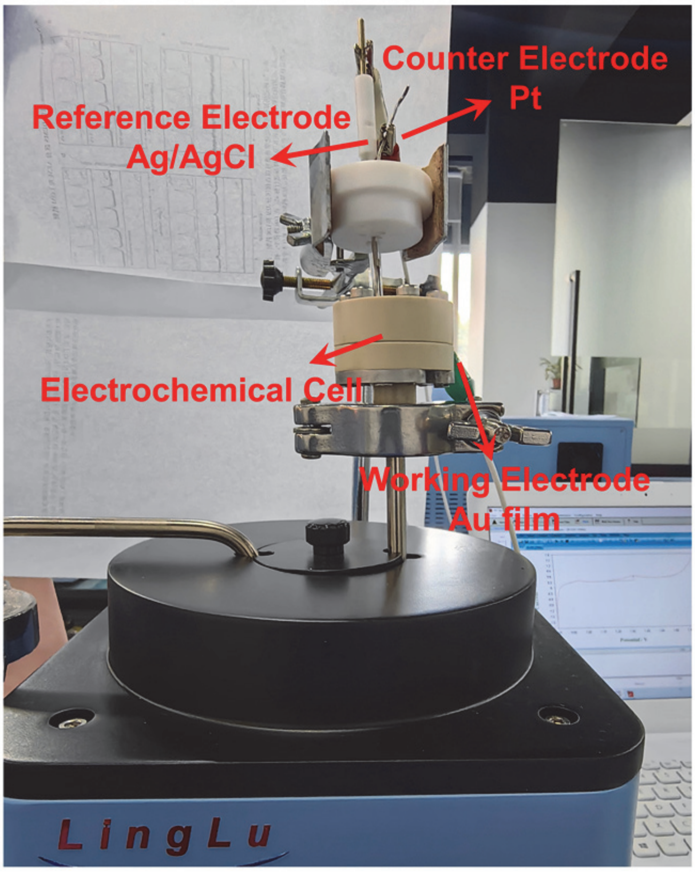

Supplementary Fig. 28 Equipment for in-situ differential electrochemical mass spectroscopy test equipment.

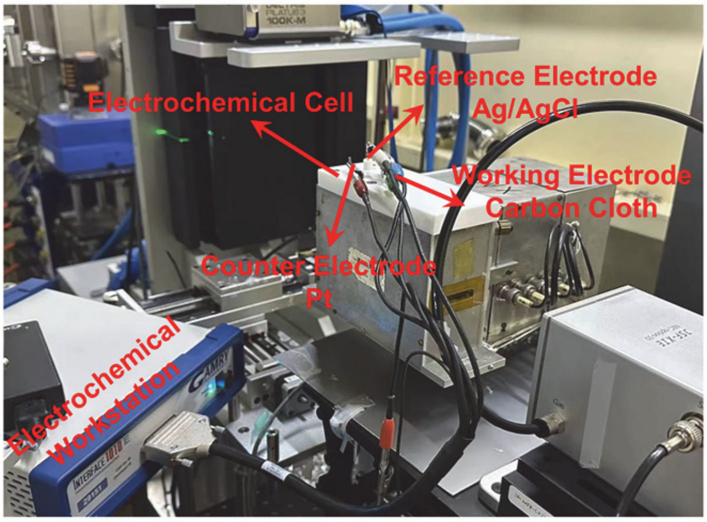

Supplementary Fig. 29 Equipment for in-situ X-ray absorption spectra test equipment.

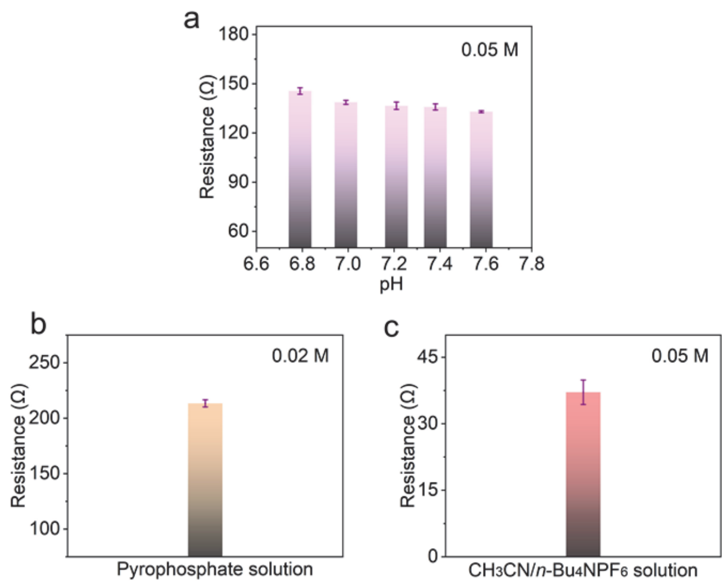

Supplementary Fig. 30 (a) The resistance values of PBS solutions with different pH values. (b) The resistance value of pyrophosphate solution. (c) The resistance value of CH_3CN/*n*-Bu_4NPF_6 solution. The error bars were the standard deviations of three repeated measurements.
